# Crystal structure of bis­[di­hydro­bis­(pyrazol-1-yl)borato-κ^2^ *N* ^2^,*N* ^2′^](1,10-phenanthroline-κ^2^ *N*,*N*′)zinc(II)

**DOI:** 10.1107/S2056989019009289

**Published:** 2019-07-04

**Authors:** Sascha Ossinger, Christian Näther, Felix Tuczek

**Affiliations:** a Institut für Anorganische Chemie, Christian-Albrechts-Universität Kiel, Max-Eyth Str. 2, D-24118 Kiel, Germany

**Keywords:** crystal structure, model substance for Fe^II^ di­hydro­bis­(pyrazol-1-yl)borate phenanthroline, Zn^II^

## Abstract

The asymmetric unit of the title compound, [Zn(C_6_H_8_N_4_B)_2_(C_12_H_8_N_2_)], comprises one half of a Zn^II^ cation (site symmetry 2), one di­hydro­bis­(pyrazol-1-yl)borate ligand in a general position, and one half of a phenanthroline ligand, the other half being completed by twofold rotation symmetry. The Zn^II^ cation is coordinated in form of a slightly distorted octa­hedron by the N atoms of a phenanthroline ligand and by two pairs of N atoms of symmetry-related di­hydro­bis­(pyrazol-1-yl)borate ligands. The discrete complexes are arranged into columns that elongate in the *c-*axis direction with a parallel alignment of the phenanthroline ligands, indicating weak π–π inter­actions.

## Abstract 2

In the crystal structure of the title compound, the Zn^II^ cation is octa­hedrally coordinated by the N atoms of a chelating phenanthroline ligand and the N atoms of two symmetry-related di­hydro­bis­(pyrazol-1-yl)borate ligands into discrete complexes.

## Chemical context   

Spin-crossover transition-metal complexes (3*d* ^4^–3*d* ^7^) continue to be a fascinating class of functional materials in the field of coordination chemistry and have the potential to play a significant role in electronic data storage or in spintronics (Gütlich *et al.*, 2013 ▸; Halcrow, 2013 ▸). Transitions between the diamagnetic low spin state (*S* = 0 for Fe^II^) and the paramagnetic high-spin state (*S* = 2 for Fe^II^) of such complexes can be induced by stimuli such as temperature or light. In most cases, spin-crossover complexes are based on octa­hedral [Fe^II^N_6_] coordination environments with chelating or mono-coordinating nitro­gen donor ligands. From all metal ions and ligands leading to spin-crossover complexes, the Fe^II^/nitro­gen ligand combination leads to the greatest changes in metal–ligand bond lengths between the two spin states and so far to the longest-lived photochemical excited spin state (Halcrow, 2007 ▸). Since the beginning of this research area some several decades ago, this field has been directed towards applications using the change of the magnetic and electronic properties of the spin-crossover compounds associated with the spin transition. Regarding applications, it might be advantageous to deposit spin-crossover complexes as thin films on substrates. This can be achieved by different methods of which physical vapour deposition is the most practicable because the formation of solvates can be ruled out. In this context, we have deposited various complexes with organoborate ligands mainly based on di­hydro­bis­(pyrazol-1-yl)borate on different substrates (Naggert *et al.*, 2011 ▸, 2015 ▸; Ossinger *et al.*, 2017 ▸; Gopakumar *et al.*, 2012 ▸; Kipgen *et al.*, 2018 ▸).

In the course of this project we became inter­ested in the well-known iron spin-crossover complex [Fe(H_2_B(pz)_2_)_2_(phen)] ((H_2_B(pz)_2_)_2_ = bis­(di­hydro­bis­(pyrazol-1-yl)borate); phen = 1,10-phenanthroline). To make conclusions regarding the behaviour of [Fe(H_2_B(pz)_2_)_2_(phen)] on substrates such as, for example, graphene, quantum-chemical calculations using the *xTB* program (Grimme *et al.*, 2017 ▸; Bannwarth *et al.*, 2019 ▸) are useful. We are especially inter­ested in structural details of the high-spin state, but unfortunately for iron(II) complexes the geometry optimization always leads to the low-spin state. To overcome this problem, corresponding compounds with Zn^II^ can be used in the calculation, because their geometry is close to that of Fe^II^ compounds in the high-spin state. This approach is beneficial because the calculation of diamagnetic compounds is simpler and, in addition, diamagnetic compounds can easily be investigated by NMR spectroscopy. Therefore, Zn^II^ complexes are often used as model systems for high-spin iron(II) complexes (Seredyuk *et al.*, 2007 ▸; Schenker *et al.*, 2001 ▸). The ionic radii (Shannon, 1976 ▸) for Zn^II^ cations (3*d* ^10^, ^1^ *S*) are nearly the same as for Fe^II^ cations in the high-spin state (3*d* ^6^, ^5^ *T*_2_), frequently leading to the formation of isotypic compounds.

With these consideration in mind, [Zn(H_2_B(pz)_2_)_2_(phen)] was synthesized, crystallized and investigated by single crystal X-ray diffraction. The X-ray powder pattern revealed that a pure compound was obtained (see Fig. S1 in the supporting information) that is suitable for physical vapour deposition, in analogy to the Fe^II^ analogue (Naggert *et al.*, 2011 ▸, 2015 ▸; Ossinger *et al.*, 2017 ▸). Comparison of the infrared spectra from the bulk and vacuum-deposited Zn^II^ compound shows identical vibrational modes, proving that no decomposition takes place during deposition (Fig. S2).
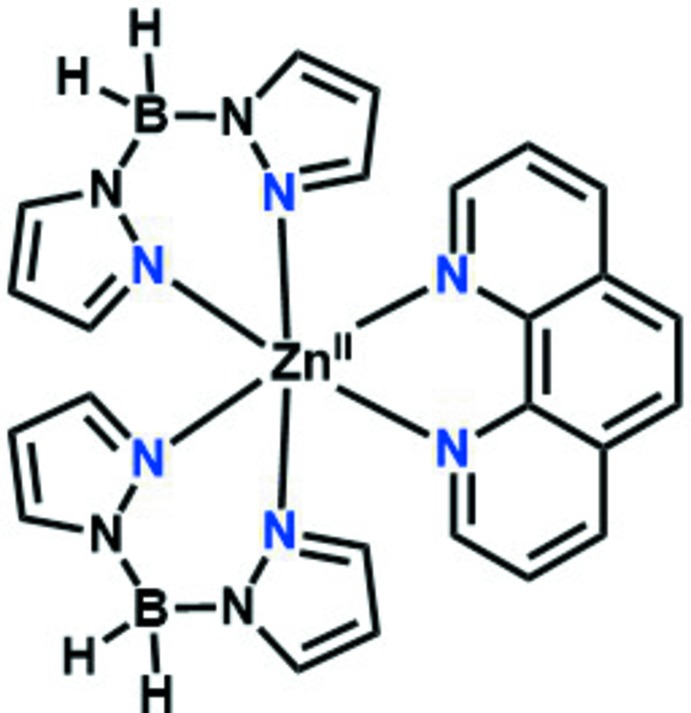


## Structural commentary   

**Figure 1:**
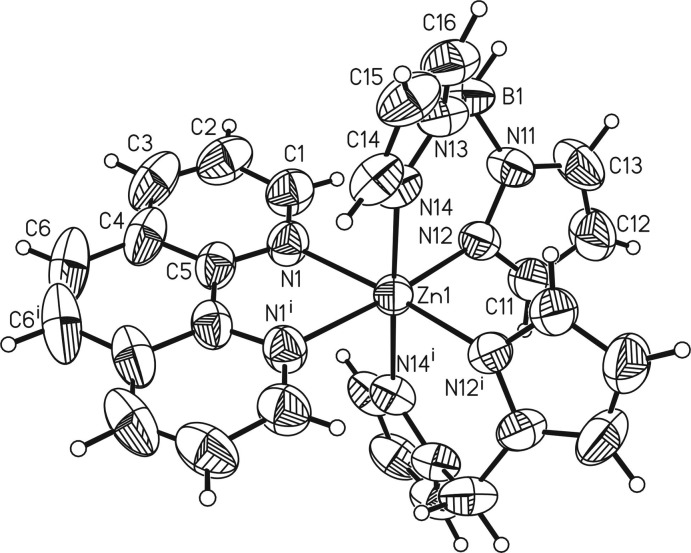
Mol­ecular structure of the title compound with the atom labelling and displacement ellipsoids drawn at the 50% probability level. [Symmetry code: (i) −*x* + 1, *y*, −*z* + 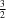.]

[Zn(H_2_B(pz)_2_)_2_(phen)] is isotypic with the Fe^II^ analogue (Real *et al.*, 1997 ▸). The asymmetric unit of the title compound consists of one di­hydro­bis­(pyrazol-1-yl)borate ligand, one half of a Zn^II^ cation located on a twofold rotation axis and one half of a phenanthroline ligand, the other half being completed by application of twofold rotation symmetry. The Zn^II^ cation is coordinated by the N atoms of the chelating phenanthroline ligand and by two pairs of N atoms of two symmetry-related di­hydro­bis­(pyrazol-1-yl)borate ligands, leading to a slightly distorted octa­hedral coordination environment (Fig. 1 ▸), as shown by the different bond lengths and angles deviating from ideal values (Table 1 ▸). The Zn—N bond lengths involving the di­hydro­bis­(pyrazol-1-yl)borate ligand are 2.1454 (18) and 2.1705 (18) Å and thus are significantly shorter than those to the phenanthroline ligand of 2.2101 (19) Å. The planes of the five-membered rings of the di­hydro­bis­(pyrazol-1-yl)borate ligand are rotated with respect to each other by 44.4 (2)°.

## Supra­molecular features   

**Figure 2:**
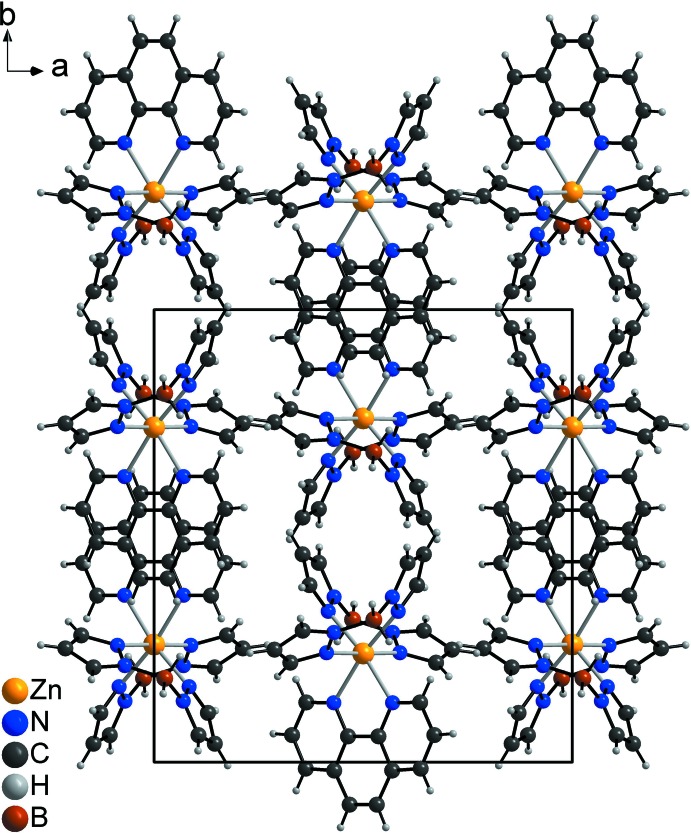
Crystal structure of the title compound in a view along the *c* axis.

**Figure 3:**
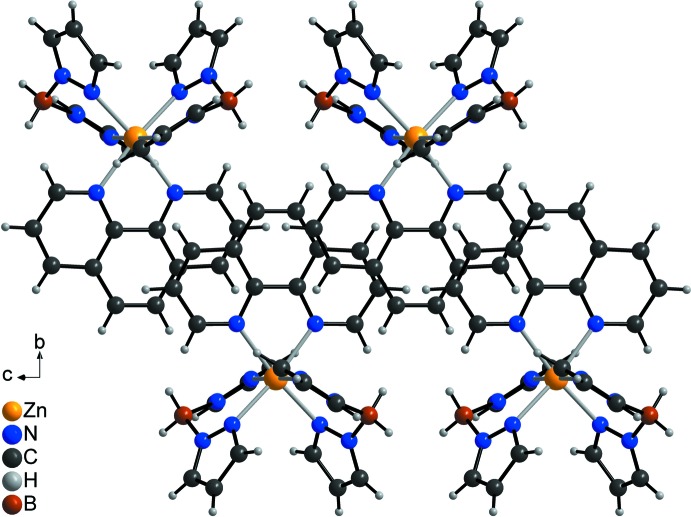
Parts of the crystal structure of the title compound emphasizing the arrangement of the phenanthroline ligands.

In the crystal structure of the title compound, the discrete complexes are arranged into columns that elongate in the *c*-axis direction (Fig. 2 ▸). Within these columns, the phenanthroline ligands are parallel but shifted relative to each other (Fig. 3 ▸). The shortest distance between two parallel phenanthroline planes amounts to 3.9341 (11) Å, indicative of weak π–π inter­actions.

## Database survey   

There are already 17 crystal structures of iron complexes with di­hydro­bis­(pyrazol-1-yl)borate and different co-ligands reported in the literature, which includes [Fe(H_2_B(pz)_2_)_2_(phen)] and [Fe(H_2_B(pz)_2_)_2_(2,2′-bipy)] (Real *et al.*, 1997 ▸; Thompson, *et al.*, 2004 ▸) as the most well-known complexes. In the others, the co-ligand is exchanged by annelated bipyridyl ligands (Kulmaczewski *et al.*, 2014 ▸), various modified di­aryl­ethene ligands (Nihei *et al.*, 2013 ▸; Milek *et al.*, 2013 ▸; Mörtel *et al.*, 2017 ▸), 4,7-dimethyl-phenanthroline (Naggert *et al.*, 2015 ▸), di­methyl­bipyridine derivatives substituted in the 5,5′ position (Xue *et al.*, 2018 ▸), diaminobipyridine (Luo *et al.*, 2016 ▸) and chiral *(R)/(S)*-4,5-pinenepyridyl-2-pyrazine ligands (Ru *et al.*, 2017 ▸). In all of these complexes, the Fe^II^ cations are coordinated by three bidentate chelate ligands in an octa­hedral environment and show spin-crossover behaviour. Moreover, the structure of the synthetic inter­mediate used for the preparation of the Fe phenanthroline complex, [Fe(H_2_B(pz)_2_)_2_(MeOH)_2_], has also been published (Ossinger *et al.*, 2016 ▸).

To the best of our knowledge, no zinc complex with the di­hydro­bis­(pyrazol-1-yl)borate ligand and additional co-ligands has been reported in the literature. So far only the complex [Zn(H_2_B(pz)_2_)_2_] (Reger *et al.*, 2000 ▸) and four related compounds with di­hydro­bis­(pyrazol-1-yl)borate modified by different substituents at the pyrazole unit have been reported (Rheingold *et al.*, 2000 ▸; Agrifoglio & Capparelli, 2005 ▸; Dias & Gorden, 1996 ▸). In all of these complexes, the Zn^II^ cations are tetra­hedrally coordinated by two bidentate organoborate ligands based on di­hydro­bis­(pyrazol-1-yl)borate. There are other zinc complexes supported by the tripodal hydro­tris(pyrazol-1-yl)borate ligand (Nakata *et al.*, 1995 ▸) with various substituents at the pyrazole unit forming different solvates (Reger *et al.*, 2000 ▸; Kitano *et al.*, 2003 ▸; Lobbia *et al.*, 1997 ▸; Yang *et al.*, 1997 ▸; Calvo & Vahrenkamp, 2006 ▸; Janiak *et al.*, 2000 ▸; Looney *et al.*, 1995 ▸; Bats & Guo, 2014 ▸). In the zinc complexes, the metal cations are in each case coordinated by two tripodal ligands in an octa­hedral coordination environment.

## Synthesis and crystallization   

1*H* **-**pyrazole, potassium tetra­hydro­borate, zinc perchlorate hexa­hydrate and 1,10-phenanthroline were purchased and used without further purification. Solvents were purchased and purified by distilling over conventional drying agents. K[H_2_B(pz)_2_] and [Zn(H_2_B(pz)_2_)_2_(phen)] were synthesized according to previously reported procedures (Naggert *et al.*, 2011 ▸, 2015 ▸; Ossinger *et al.*, 2016 ▸, 2017 ▸).

**Synthesis of [Zn(H_2_B(pz)_2_)_2_(phen)]:** To a solution of Zn(ClO_4_)_2_·6H_2_O (746 mg, 2.00 mmol) in methanol (10 ml) a solution of K[H_2_B(pz)_2_] (744 mg, 4.00 mmol) in methanol (10 ml) was added. After 15 min of stirring, precipitated KClO_4_ was removed by filtration. To the filtrate a solution of 1,10-phenanthroline (361 mg, 2.00 mmol) in methanol (10 ml) was added dropwise, leading to the formation of a colourless precipitate. The mixture was stirred for another hour at room temperature and the precipitate was filtered off, washed with methanol (5 ml) and filtered again by suction filtration (30 min). Yield: 142 mg (263 µmol, 13% based on Zn(ClO_4_)_2_·6H_2_O).

**Elemental analysis** calculated for C_24_H_24_B_2_ZnN_10_: C 53.42, H 4.48, N 25.96%, found: C 53.39, H 4.47, N 25.98%.

**HRESI–MS(+)(CHCl_3_ + MeOH):** *m*/*z* (%) = [*M* − H_2_B(pz)_2_]^+^ calculated 391.08155, found 391.08061 (5).

**^1^H NMR (400 MHz, CDCl_3_):** *δ* (ppm) = 9.21 (*dd*, *J* = 4.3Hz, 1.7Hz, 2H, phen-H^4^), 8.27 (*dd*, *J* = 8.1Hz, 1.7Hz, 2H, phen-H^4^), 7.81 (*s*, 2H, phen-H^7^), 7.73 (*dd*, *J* = 2.2Hz, 0.5Hz, 4H, pyrazolyl-H^5^), 7.65 (*dd*, *J* = 8.1Hz, 4.3Hz, 2H, phen-H^3^), 7.57 (*d*, *J* = 1.9Hz, 4H, pyrazolyl-H^3^), 6.28 (*t*, *J* = 2.1Hz, 4H, pyrazolyl-H^4^), 3.78 (*br. d*, *J* = 127.9Hz, 4H, B-H).

**^13^C{^1^H} NMR (100 MHz, CDCl_3_):** *δ*/ppm = 150.5 (CH, phen-C^2^), 146.39 (C_q_, phen-C^6^), 140.31 (CH, pyrazolyl-C^3^), 136.93 (CH, pyrazolyl-C^5^), 136.14 (CH, phen-C^4^), 128.8 (C_q_, phen-C^5^), 126.68 (CH, phen-C^7^), 123.24 (CH, phen-C^3^), 105.13 (CH, pyrazolyl-C^4^).

**^1^B NMR (128 MHz, CDCl_3_):** *δ*/ppm = −8.43 (*br. s* (*t*), 1B).

**IR (ATR, 298 K):** ν/cm^−1^ = 3134, 3118, 3073, 3060 [*w*, *ν* (=C—H)], 2464, 2438, 2397, 2356 [*m*, *ν*_asym._ (–BH_2_)], 2309, 2295 [*m*, *ν*_sym._ (–BH_2_)], 1719 (*w*), 1625 (*w*), 1595 (*w*), 1578 (*w*), 1515 (*m*). 1494 (*m*), 1425 (*m*), 1399 (*m*), 1347 (*w*), 1321 (*w*), 1294 (*m*), 1266 (*w*), 1213 (*m*), 1200 (*m*), 1186 (*m*), 1172 (*m*), 1160 (*s*), 1137 (*w*), 1098 (*w*), 1090 (*w*), 1064 (*m*), 1049 (*s*), 1011 (*w*), 978 (*m*), 960 (*w*), 921 (*w*), 900 (*w*), 882 (*m*), 866 (*w*), 843 (*m*), 806 (*w*), 782 (*s*), 747 (*s*), 727 (*s*), 717 (*m*), 678 (*m*), 649 (*m*), 637 (*s*), 623 (*m*), 480 (*w*), 437 (*w*), 421 (*w*).

**Raman (Bulk, 298 K):** ν (cm^−1^) = 3134, 3115, 3088, 3074, 3061, 3028, 2997 [*m*, *ν* (=C—H)], 2472, 2447, 2397, 2359 [*w*, *ν*_asym._ (–BH_2_)], 2310, 2297 [*w*, *ν*_sym._ (–BH_2_)], 1626 (*w*), 1605 (*w*), 1589 (*w*), 1516 (*w*), 1452 (*m*), 1419 (*s*), 1408 (*m*), 1350 (*w*), 1308 (*m*), 1296 (*m*), 1213 (*m*), 1163 (*w*), 1138 (*w*), 1097 (*w*), 1057 (*w*), 1045 (*m*), 1012 (*w*), 980 (*w*), 924 (*w*), 727 (*m*), 559 (*w*), 422 (*w*), 411 (*w*).

**UV/Vis (KBr, 298 K):** λ_*max*_ (nm) = 204, 230, 274, 298, 332, 448–600 (*br*), 600–650 (*br*).

**Crystallization:** Single crystals of [Zn(H_2_B(pz)_2_)_2_(phen)] were obtained under synthetic conditions as described above. After the precipitate was filtered off and washed with methanol, the mother liquor was stored at 278 K. After a few days colourless block-like single crystals had formed.

**Experimental details:** NMR spectra were recorded in deuterated solvents with a Bruker Avance 400 spectrometer operating at a ^1^H frequency of 400 MHz, a ^13^C frequency of 100 MHz and a ^11^B frequency of 128 MHz. They were referenced to the residual protonated solvent signal [^1^H: *δ*(CDCl_3_) = 7.26 ppm], the solvent signal [^13^C: *δ*(CDCl_3_) = 77.16 ppm] or an external standard (^11^B:BF_3_·Et_2_O) (Gottlieb *et al.*, 1997 ▸; Fulmer *et al.*, 2010 ▸). Signals were assigned with the help of DEPT-135 and two-dimensional correlation spectra (^1^H,^1^H-COSY, ^1^H,^13^C-HSQC, ^1^H,^13^C-HMBC). Signal multiplicities are abbreviated as *s* (singlet), *d* (doublet), *t* (triplet), *m* (multiplet) and *br*. (broad signal). Elemental analyses were performed using a vario MICRO cube CHNS element analyser from Elementar. Samples were burned in sealed tin containers by a stream of oxygen. High-resolution ESI mass spectra were recorded on a ThermoFisher Orbitrap spectrometer. IR spectra were recorded on a Bruker Alpha-P ATR-IR Spectrometer. Signal intensities are marked as *s* (strong), *m* (medium), *w* (weak) and *br*. (broad). For FT–Raman spectroscopy, a Bruker RAM II −1064 FT–Raman Module, a R510-N/R Nd:YAG-laser (1046 nm, up to 500 mW) and a D418-T/R liquid-nitro­gen-cooled, highly sensitive Ge detector or a Bruker IFS 66 with a FRA 106 unit and a 35mW NdYAG-LASER (1064 nm) was used. XRPD experiments were performed with a Stoe Transmission Powder Diffraction System (STADI P) with Cu K_*α*_ radiation (λ = 1.5406 Å) that is equipped with position-sensitive detectors (Mythen-K1). UV/vis spectra were recorded with a Cary 5000 spectrometer in transmission geometry.

## Refinement   

Crystal data, data collection and structure refinement details are summarized in Table 2 ▸. The H atoms were positioned with idealized geometry (C—H = 0.93 Å) and were refined with *U*_iso_(H) = 1.2*U*_eq_(C) using a riding model. The B—H hydrogen atoms were located in a difference-Fourier map. Their bond lengths were set to ideal values (B—H = 0.97 Å) and finally they were refined with *U*_iso_(H) = 1.5*U*_eq_(B) using a riding model.

## Supplementary Material

Crystal structure: contains datablock(s) I. DOI: 10.1107/S2056989019009289/wm5509sup1.cif

Structure factors: contains datablock(s) I. DOI: 10.1107/S2056989019009289/wm5509Isup2.hkl

Figure S1. Experimental XRPD pattern of [Zn(H2B(pz)2)2(phen)] measuered at rt (a) as well as simulated XRPD pattern calculated from the single crystal structure (b) (293 K) in comparison with the calculated pattern for the isostructural high-spin complex [Fe(H2B(pz)2)2(phen)] (c) retrieved from literature (Real et al., 1997). DOI: 10.1107/S2056989019009289/wm5509sup3.jpg

Figure S2. Fourier transform infrared (FT-IR) spectra of bulk material (black line) and vacuum-deposited material (red line) of the zinc complex at rt. DOI: 10.1107/S2056989019009289/wm5509sup4.jpg

CCDC reference: 1937083

Additional supporting information:  crystallographic information; 3D view; checkCIF report

## Figures and Tables

**Table 1:** Selected geometric parameters (Å, °)

Zn1—N12	2.1454 (18)	Zn1—N1	2.2101 (19)
Zn1—N14	2.1704 (18)		
			
N12—Zn1—N12^i^	91.24 (10)	N14—Zn1—N1	89.34 (7)
N12—Zn1—N14	90.43 (7)	N12—Zn1—N1^i^	171.59 (7)
N12—Zn1—N14^i^	88.55 (7)	N14—Zn1—N1^i^	91.83 (7)
N14—Zn1—N14^i^	178.54 (11)	N1—Zn1—N1^i^	75.01 (11)
N12—Zn1—N1	96.92 (7)		

Symmetry code: (i) 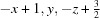.

**Table 2:** Experimental details

Crystal data	
Chemical formula	[Zn(C_6_H_8_BN_4_)_2_(C_12_H_8_N_2_)]
*M*_r_	539.52
Crystal system, space group	Monoclinic, *C*2/*c*
Temperature (K)	293
*a*, *b*, *c* (Å)	17.4591 (10), 16.0990 (7), 10.6076 (6)
β (°)	121.533 (4)
*V* (Å^3^)	2541.3 (3)
*Z*	4
Radiation type	Mo *K*α
μ (mm^−1^)	1.00
Crystal size (mm)	0.13 × 0.10 × 0.06

Data collection	
Diffractometer	Stoe IPDS2
Absorption correction	Numerical (*X-RED* and *X-SHAPE*; Stoe & Cie, 2008 ▸)
*T*_min_, *T*_max_	0.805, 0.911
No. of measured, independent and observed [*I* > 2σ(*I*)] reflections	10361, 2765, 2359
*R*_int_	0.031
(sin θ/λ)_max_ (Å^−1^)	0.639

Refinement	
*R*[*F* ^2^ > 2σ(*F* ^2^)], *wR*(*F* ^2^), *S*	0.039, 0.089, 1.08
No. of reflections	2765
No. of parameters	168
H-atom treatment	H-atom parameters constrained
Δρ_max_, Δρ_min_ (e Å^−3^)	0.22, −0.22

Computer programs: *X-AREA* (Stoe & Cie, 2008 ▸), *SHELXS97* (Sheldrick, 2008 ▸), *SHELXL2014* (Sheldrick, 2015 ▸), *DIAMOND* (Brandenburg, 1999 ▸) and *publCIF* (Westrip, 2010 ▸).

## supplementary crystallographic information

### Crystal data

**Table:**

[Zn(C_6_H_8_BN_4_)_2_(C_12_H_8_N_2_)]	*F*(000) = 1112
*M**_r_* = 539.52	*D*_x_ = 1.410 Mg m^−^^3^
Monoclinic, *C*2/*c*	Mo *K*α radiation, λ = 0.71073 Å
*a* = 17.4591 (10) Å	Cell parameters from 10361 reflections
*b* = 16.0990 (7) Å	θ = 2.3–27.0°
*c* = 10.6076 (6) Å	µ = 1.00 mm^−^^1^
β = 121.533 (4)°	*T* = 293 K
*V* = 2541.3 (3) Å^3^	Block, colourless
*Z* = 4	0.13 × 0.10 × 0.06 mm

### Data collection

**Table:**

Stoe IPDS-2 diffractometer	2359 reflections with *I* > 2σ(*I*)
ω scans	*R*_int_ = 0.031
Absorption correction: numerical (X-RED and X-SHAPE; Stoe & Cie, 2008)	θ_max_ = 27.0°, θ_min_ = 2.3°
*T*_min_ = 0.805, *T*_max_ = 0.911	*h* = −21→22
10361 measured reflections	*k* = −20→16
2765 independent reflections	*l* = −13→13

### Refinement

**Table:**

Refinement on *F*^2^	0 restraints
Least-squares matrix: full	Hydrogen site location: inferred from neighbouring sites
*R*[*F*^2^ > 2σ(*F*^2^)] = 0.039	H-atom parameters constrained
*wR*(*F*^2^) = 0.089	*w* = 1/[σ^2^(*F*_o_^2^) + (0.0445*P*)^2^ + 0.6977*P*] where *P* = (*F*_o_^2^ + 2*F*_c_^2^)/3
*S* = 1.08	(Δ/σ)_max_ < 0.001
2765 reflections	Δρ_max_ = 0.22 e Å^−^^3^
168 parameters	Δρ_min_ = −0.22 e Å^−^^3^

### Special details

**Table:**

Geometry. All esds (except the esd in the dihedral angle between two l.s. planes) are estimated using the full covariance matrix. The cell esds are taken into account individually in the estimation of esds in distances, angles and torsion angles; correlations between esds in cell parameters are only used when they are defined by crystal symmetry. An approximate (isotropic) treatment of cell esds is used for estimating esds involving l.s. planes.

### Fractional atomic coordinates and isotropic or equivalent isotropic displacement parameters (Å^2^)

**Table:**

	*x*	*y*	*z*	*U*_iso_*/*U*_eq_	
Zn1	0.5000	0.75993 (2)	0.7500	0.04673 (13)	
N1	0.42976 (13)	0.86884 (12)	0.6097 (2)	0.0560 (5)	
C1	0.36392 (18)	0.86814 (18)	0.4692 (3)	0.0689 (7)	
H1	0.3393	0.8173	0.4246	0.083*	
C2	0.3299 (2)	0.9403 (2)	0.3851 (4)	0.0835 (9)	
H2	0.2851	0.9372	0.2855	0.100*	
C3	0.3626 (2)	1.0144 (2)	0.4497 (4)	0.0912 (10)	
H3	0.3399	1.0630	0.3950	0.109*	
C4	0.4307 (2)	1.01866 (16)	0.5991 (4)	0.0762 (8)	
C5	0.46363 (16)	0.94306 (13)	0.6741 (3)	0.0556 (5)	
C6	0.4676 (3)	1.09407 (18)	0.6787 (5)	0.1086 (14)	
H6	0.4458	1.1445	0.6300	0.130*	
N11	0.43035 (14)	0.63487 (13)	0.4888 (2)	0.0595 (5)	
N12	0.41630 (13)	0.66673 (11)	0.5937 (2)	0.0518 (4)	
C11	0.37140 (18)	0.60880 (16)	0.6182 (3)	0.0642 (6)	
H11	0.3534	0.6138	0.6863	0.077*	
C12	0.3549 (2)	0.54065 (19)	0.5298 (4)	0.0870 (9)	
H12	0.3241	0.4925	0.5248	0.104*	
C13	0.3939 (2)	0.55958 (19)	0.4513 (4)	0.0830 (9)	
H13	0.3950	0.5251	0.3819	0.100*	
B1	0.4746 (2)	0.6851 (2)	0.4199 (3)	0.0690 (8)	
H1A	0.4381	0.7334	0.3693	0.083*	
H1B	0.4782	0.6511	0.3476	0.083*	
N13	0.57014 (14)	0.71333 (13)	0.5411 (2)	0.0580 (5)	
N14	0.58774 (13)	0.75821 (12)	0.6621 (2)	0.0546 (4)	
C14	0.67425 (17)	0.78014 (16)	0.7300 (3)	0.0631 (6)	
H24	0.7046	0.8106	0.8173	0.076*	
C15	0.71221 (18)	0.75136 (19)	0.6524 (3)	0.0732 (7)	
H15	0.7710	0.7589	0.6753	0.088*	
C16	0.64476 (18)	0.70945 (19)	0.5346 (3)	0.0695 (7)	
H16	0.6497	0.6825	0.4617	0.083*	

### Atomic displacement parameters (Å^2^)

**Table:**

	*U*^11^	*U*^22^	*U*^33^	*U*^12^	*U*^13^	*U*^23^
Zn1	0.0499 (2)	0.0432 (2)	0.04314 (19)	0.000	0.02155 (15)	0.000
N1	0.0552 (11)	0.0514 (11)	0.0531 (11)	0.0032 (9)	0.0225 (9)	0.0053 (8)
C1	0.0653 (16)	0.0721 (16)	0.0568 (15)	0.0089 (13)	0.0232 (13)	0.0111 (12)
C2	0.0744 (19)	0.099 (2)	0.0705 (18)	0.0207 (17)	0.0335 (16)	0.0335 (17)
C3	0.081 (2)	0.079 (2)	0.115 (3)	0.0242 (17)	0.052 (2)	0.051 (2)
C4	0.0742 (18)	0.0522 (14)	0.112 (2)	0.0121 (13)	0.0552 (18)	0.0250 (14)
C5	0.0550 (13)	0.0459 (12)	0.0735 (15)	0.0046 (10)	0.0390 (12)	0.0063 (10)
C6	0.104 (3)	0.0439 (14)	0.174 (4)	0.0090 (15)	0.070 (3)	0.0195 (17)
N11	0.0640 (12)	0.0682 (12)	0.0513 (11)	−0.0061 (10)	0.0338 (10)	−0.0125 (9)
N12	0.0583 (11)	0.0524 (10)	0.0486 (10)	−0.0039 (8)	0.0307 (9)	−0.0052 (8)
C11	0.0742 (17)	0.0613 (14)	0.0657 (15)	−0.0147 (12)	0.0425 (14)	−0.0092 (11)
C12	0.110 (3)	0.0628 (16)	0.108 (2)	−0.0286 (17)	0.070 (2)	−0.0235 (16)
C13	0.094 (2)	0.0747 (18)	0.088 (2)	−0.0200 (16)	0.0525 (19)	−0.0387 (16)
B1	0.0674 (18)	0.098 (2)	0.0443 (14)	−0.0064 (16)	0.0308 (14)	−0.0032 (14)
N13	0.0588 (11)	0.0716 (12)	0.0495 (10)	0.0034 (9)	0.0324 (9)	0.0081 (9)
N14	0.0524 (10)	0.0625 (11)	0.0484 (9)	−0.0004 (9)	0.0260 (8)	0.0083 (8)
C14	0.0559 (13)	0.0688 (15)	0.0584 (13)	−0.0027 (11)	0.0256 (12)	0.0166 (11)
C15	0.0540 (13)	0.095 (2)	0.0748 (16)	0.0067 (14)	0.0365 (13)	0.0276 (15)
C16	0.0663 (16)	0.0879 (18)	0.0663 (16)	0.0139 (14)	0.0430 (15)	0.0181 (13)

### Geometric parameters (Å, º)

**Table:**

Zn1—N12	2.1454 (18)	N11—N12	1.358 (2)
Zn1—N12^i^	2.1454 (18)	N11—B1	1.541 (4)
Zn1—N14	2.1704 (18)	N12—C11	1.328 (3)
Zn1—N14^i^	2.1705 (18)	C11—C12	1.372 (4)
Zn1—N1	2.2101 (19)	C11—H11	0.9300
Zn1—N1^i^	2.2101 (19)	C12—C13	1.356 (4)
N1—C1	1.323 (3)	C12—H12	0.9300
N1—C5	1.350 (3)	C13—H13	0.9300
C1—C2	1.394 (4)	B1—N13	1.549 (4)
C1—H1	0.9300	B1—H1A	0.9700
C2—C3	1.347 (5)	B1—H1B	0.9700
C2—H2	0.9300	N13—C16	1.341 (3)
C3—C4	1.399 (5)	N13—N14	1.361 (3)
C3—H3	0.9300	N14—C14	1.337 (3)
C4—C5	1.402 (3)	C14—C15	1.379 (4)
C4—C6	1.426 (5)	C14—H24	0.9300
C5—C5^i^	1.438 (5)	C15—C16	1.365 (4)
C6—C6^i^	1.334 (8)	C15—H15	0.9300
C6—H6	0.9300	C16—H16	0.9300
N11—C13	1.329 (3)		
			
N12—Zn1—N12^i^	91.24 (10)	C13—N11—N12	109.0 (2)
N12—Zn1—N14	90.43 (7)	C13—N11—B1	128.0 (2)
N12^i^—Zn1—N14	88.55 (7)	N12—N11—B1	122.7 (2)
N12—Zn1—N14^i^	88.55 (7)	C11—N12—N11	105.83 (19)
N12^i^—Zn1—N14^i^	90.43 (7)	C11—N12—Zn1	124.95 (15)
N14—Zn1—N14^i^	178.54 (11)	N11—N12—Zn1	123.73 (14)
N12—Zn1—N1	96.92 (7)	N12—C11—C12	111.2 (2)
N12^i^—Zn1—N1	171.59 (8)	N12—C11—H11	124.4
N14—Zn1—N1	89.34 (7)	C12—C11—H11	124.4
N14^i^—Zn1—N1	91.83 (7)	C13—C12—C11	104.3 (2)
N12—Zn1—N1^i^	171.59 (7)	C13—C12—H12	127.9
N12^i^—Zn1—N1^i^	96.92 (7)	C11—C12—H12	127.9
N14—Zn1—N1^i^	91.83 (7)	N11—C13—C12	109.6 (2)
N14^i^—Zn1—N1^i^	89.34 (7)	N11—C13—H13	125.2
N1—Zn1—N1^i^	75.01 (11)	C12—C13—H13	125.2
C1—N1—C5	118.1 (2)	N11—B1—N13	110.5 (2)
C1—N1—Zn1	126.95 (18)	N11—B1—H1A	109.6
C5—N1—Zn1	114.81 (16)	N13—B1—H1A	109.6
N1—C1—C2	122.8 (3)	N11—B1—H1B	109.6
N1—C1—H1	118.6	N13—B1—H1B	109.6
C2—C1—H1	118.6	H1A—B1—H1B	108.1
C3—C2—C1	119.2 (3)	C16—N13—N14	109.1 (2)
C3—C2—H2	120.4	C16—N13—B1	126.7 (2)
C1—C2—H2	120.4	N14—N13—B1	123.6 (2)
C2—C3—C4	120.2 (3)	C14—N14—N13	106.4 (2)
C2—C3—H3	119.9	C14—N14—Zn1	128.16 (17)
C4—C3—H3	119.9	N13—N14—Zn1	123.37 (14)
C3—C4—C5	117.0 (3)	N14—C14—C15	110.5 (2)
C3—C4—C6	124.4 (3)	N14—C14—H24	124.8
C5—C4—C6	118.6 (3)	C15—C14—H24	124.8
N1—C5—C4	122.7 (3)	C16—C15—C14	105.0 (2)
N1—C5—C5^i^	117.61 (13)	C16—C15—H15	127.5
C4—C5—C5^i^	119.68 (18)	C14—C15—H15	127.5
C6^i^—C6—C4	121.61 (19)	N13—C16—C15	109.0 (2)
C6^i^—C6—H6	119.2	N13—C16—H16	125.5
C4—C6—H6	119.2	C15—C16—H16	125.5

Symmetry code: (i) −*x*+1, *y*, −*z*+3/2.
