# Supplementary figures and images for: Crystal structure of bis­[di­hydro­bis­(pyrazol-1-yl)borato-κ2 N 2,N 2′](1,10-phenanthroline-κ2 N,N′)zinc(II)
Source: Acta Crystallogr E Crystallogr Commun. 2019 Jul 4;75(Pt 8):1112–6. doi: 10.1107/S2056989019009289 (PMC6690474; doi:10.1107/S2056989019009289)

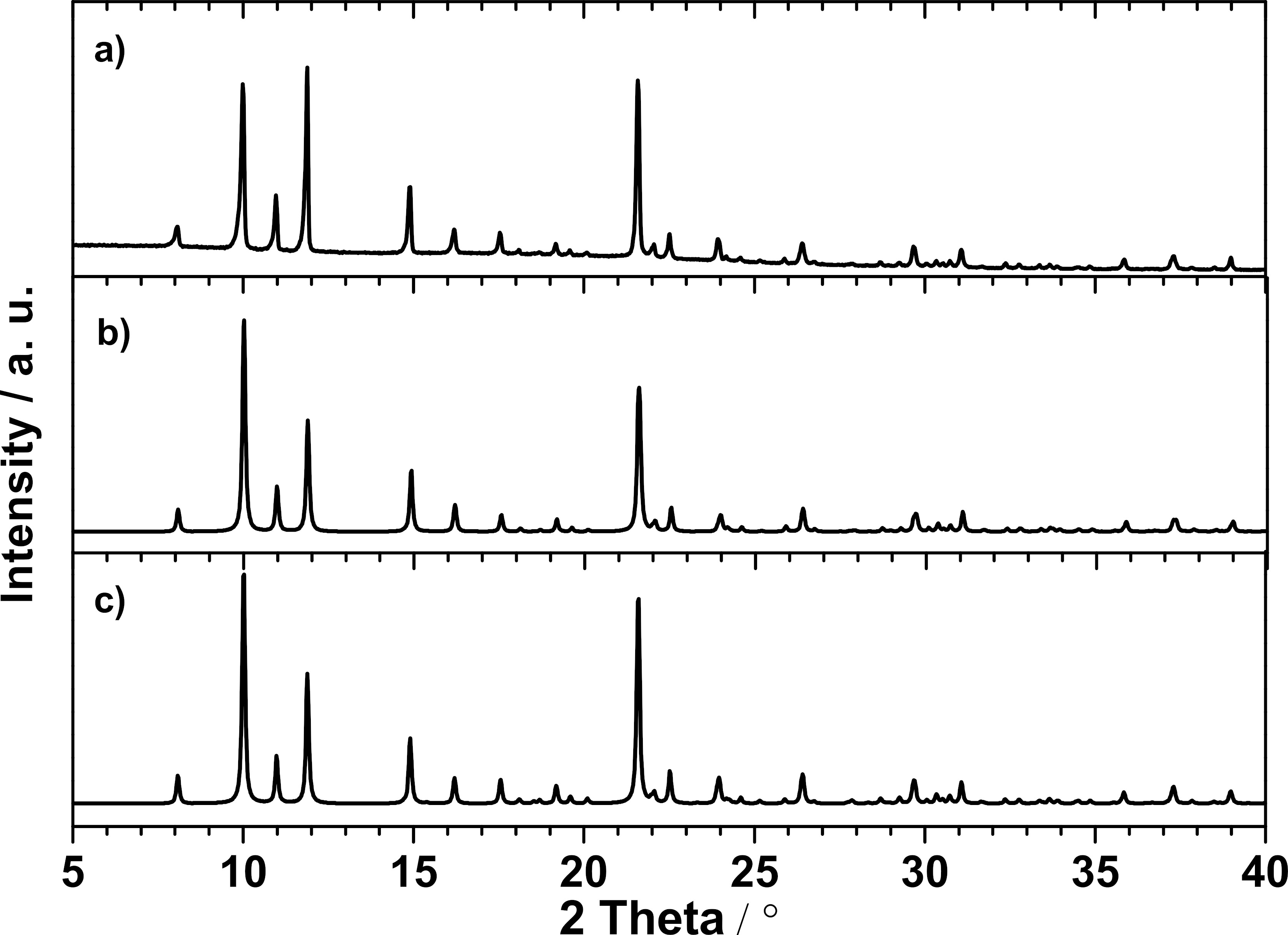

Supplement: Supplementary file 3 [file e-75-01112-sup3.jpg]

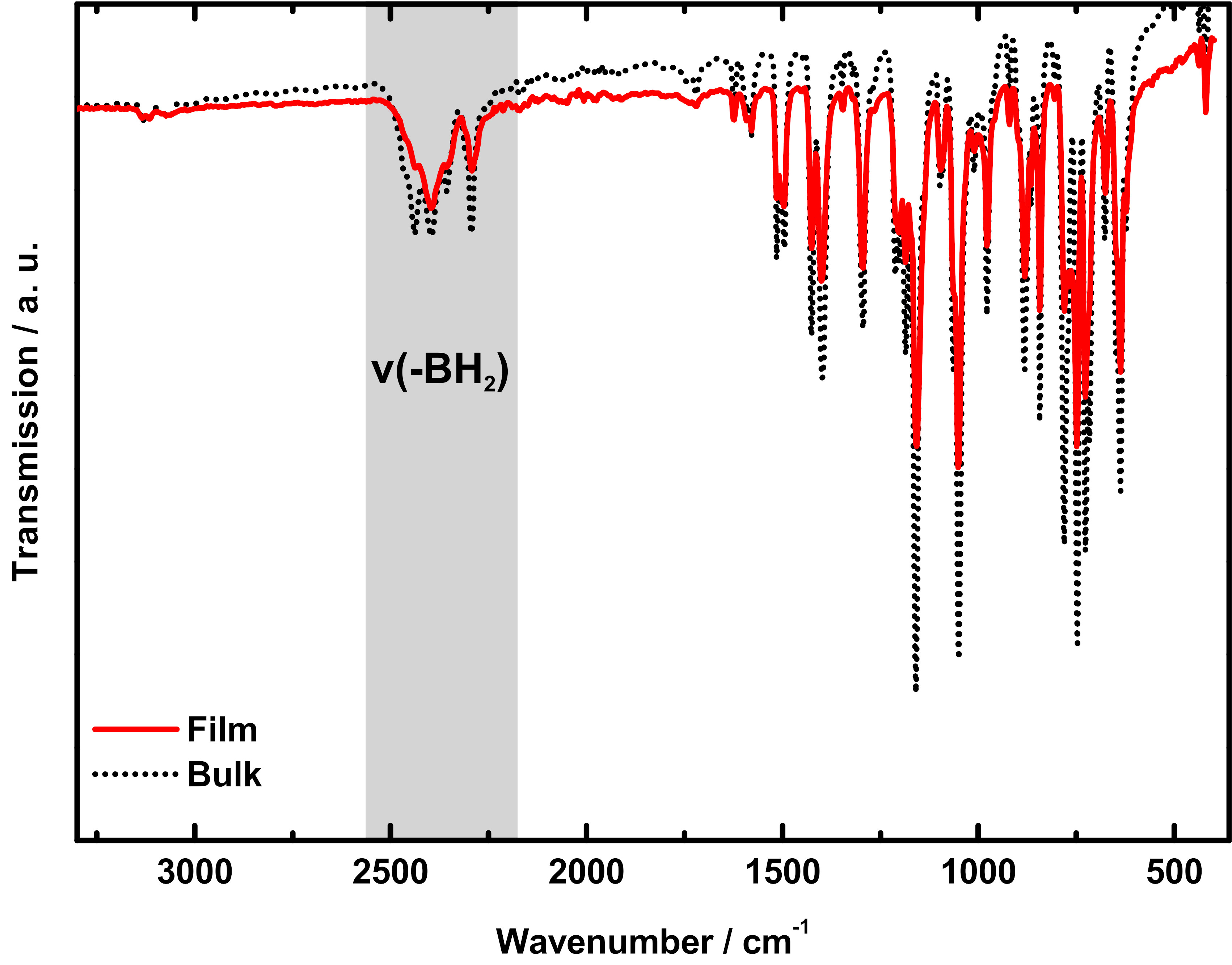

Supplement: Supplementary file 4 [file e-75-01112-sup4.jpg]
